# Genome report: genome sequence of *Phymata mystica* (Evans), an ambush bug

**DOI:** 10.1093/g3journal/jkag244

**Published:** 2026-08-28

**Authors:** Ethan Grebler, Tracy Liesenfelt, Paul K Masonick, Andrew J Mongue

**Affiliations:** Department of Entomology and Nematology, Institute of Food and Agricultural Sciences, University of Florida, 1881 Natural Area Drive, Steinmetz Hall, Gainesville, FL 32611, United States; Department of Entomology and Nematology, Institute of Food and Agricultural Sciences, University of Florida, 1881 Natural Area Drive, Steinmetz Hall, Gainesville, FL 32611, United States; Department of Natural History, Florida Museum of Natural History, McGuire Center, 3215 Hull Road, Gainesville, FL 32611, United States; Department of Entomology and Nematology, Institute of Food and Agricultural Sciences, University of Florida, 1881 Natural Area Drive, Steinmetz Hall, Gainesville, FL 32611, United States

**Keywords:** Phymatinae, Reduviidae, venomics, venom conservation, hematophagy, Hemiptera, genome assembly

## Abstract

Recent advances in sequencing technology have made the sequencing of non-model organisms significantly more streamlined and feasible. Using these technologies, we begin to address the lack of data on non-model organisms by sequencing the genome of one such species, *Phymata mystica,* an ambush bug (Hemiptera: Heteroptera: Reduviidae: Phymatinae) specialized for floral sit-and-wait style predation. Our genome assembly is 710 Mb, in which 99.7% of this sequence is assembled into 14 chromosomal scaffolds. We found that repetitive elements accounted for 58.85% of the sequence. We report 26,760 protein-coding genes in a preliminary annotation of the genome. Using these new resources, we explored both macrosynteny and gene conservation. Starting with chromosome structure, we found that *P. mystica* has a single X chromosome, unlike other well-assembled reduviids in which the X apparently split into 2 linkage groups. Exploring this new annotation, we found a number of venom proteins conserved between *P. mystica* and the other venomous Heteroptera with reference genomes, primarily serine proteases, metallopeptidases, and heteropteran venom family proteins. These results provide a new framework for the evolution of venom in this group of insects and further demonstrate the ease with which non-model species can be studied using modern genomic methods.

## Introduction

Molecular research on the ambush bugs (Reduviidae: Phymatinae), a group of early-diverging assassin bugs (Reduviidae) adapted for sit-and-wait predation, has received little attention due to historical challenges involved with the sequencing of non-model organisms’ genomes. Early research into ambush bugs was conducted mainly by European naturalists for taxonomic purposes, focusing only on a few key species (Evans 1931). The prey records of ambush bugs include a variety of insect plant pests and pollinators, making ambush bugs of potential interest for biological control and for evolutionary biology as an informative contrast to hematophagous assassin bugs (Balduf 1940). Representative species of Phymatinae have been found on every continent but Oceania and Antarctica (Masonick et al. 2017). *Phymata mystica* can be found throughout much of the southeastern region of the United States, as far north as North Carolina and as far south as the Florida Keys (Masonick and Weirauch 2020).

Like other predatory Hemiptera, ambush bugs feed by piercing their prey with their maxillary stylets and sucking out their innards. To aid in this process, ambush bugs are venomous, with their venoms serving a dual purpose: to both paralyze and to liquefy prey (Walker et al. 2017). In reduviids, venom is stored in 3 glands: the anterior main gland, the posterior main gland, and the accessory gland (Walker et al. 2018). The anterior main gland is responsible for the production of paralyzing neurotoxins, the posterior main gland is responsible for the production of digestive enzymes, and the accessory gland serves as a water reservoir hosting some “toxin-like peptides” (Walker, Mayhew, et al. 2018; Walker, Robinson, et al. 2018). The venom glands in assassin bugs have evolved from ancestral salivary glands (Walker, Robinson, et al. 2018).

The venomous proteins contained in the assassin bug sialome (proteome of the salivary gland) can be cytotoxic, hemotoxic, or neurotoxic (Walker et al. 2017; Rügen et al. 2021; Zdenek et al. 2024). Cytotoxic proteins are those possessing antimicrobial, cytolytic, and hemostasis-impairing qualities, or any qualities that disrupt cellular metabolism. Hemotoxic proteins elicit coagulation cascade activating or inhibiting, fibrinolytic, hemolytic, hypotensive, platelet aggregation activating/inhibiting, and vasodilating activity. Neurotoxic proteins elicit presynaptic and postsynaptic damage or disruption to the neuron (Zhou et al. 2022).

The sialome of predatory reduviids is primarily made up of serine proteases, whereas the venome of hematophagous reduviids is primarily made up of triabins, with very little protease content (Zdenek et al. 2024). Assassin bug venoms are anticoagulatory, leading to hematophagous species adopting this trait for its advantages for blood feeding and losing the additional painful and membrane-permeating effects of predatory assassin bug venom (Zdenek et al. 2024). The anticoagulant activity of assassin bug venoms is facilitated by several protein families. Anticoagulant activity in predatory assassin bugs is promoted by serine proteases through fibrinogen destruction; in hematophagous assassin bugs, this function is performed by lipocalins through thrombin inhibition (Zdenek et al. 2024). Anticoagulant activity in bedbugs (*Cimex lectularius*), close relatives of assassin bugs, is facilitated through apyrases (Valenzuela et al. 1996). Predatory assassin bug venoms cause pain through the cytolytic action of redulysins (Fischer et al. 2023).

Despite the wealth of knowledge for these species’ venoms, existing research has yet to explore the venoms of ambush bugs, one of the earliest diverging lineages of assassin bugs. Characterization of these insects’ venoms will add context to the evolution of venoms in the family Reduviidae. To begin adding this context, we present the genome, gene annotation, and preliminary venom ortholog analysis of *P. mystica.* Here, we generate the chromosome-level assembly and annotation of *P. mystica*'s genome, the first member of Phymatinae to be sequenced, and provide insights into the diversity and evolution of assassin bugs. Alongside the presentation of *P. mystica*'s genome and annotation, we analyze venom conservation between it and other predatory reduviids, as well as hematophagous reduviids and non-reduviid heteropteran species. These early results add to the understanding of the evolution of venoms across feeding behaviors and diversification of assassin bugs, and serve to benefit future systematic and evolutionary studies of the group.

## Materials and methods

### Collection of *P. mystica*

We sourced DNA from adult *P. mystica* collected from flowers, primarily goldenrod (*Solidago spp.*), within the Natural Area Teaching Laboratory at the University of Florida, located in Alachua County, Florida (29°38′00.2″N 82°22′04.3″W). Collection took place in early October 2024. We snap-froze live individuals by placing them directly into a −80 °C freezer, where we stored tissue until use for sequencing.

### DNA extraction and sequencing

#### Homogenization and DNA extraction

We first utilized tissue from a single male, splitting the body tissue into 2 extractions, 1 containing the head and thorax, the other containing the abdomen. These body tissues were homogenized with a Dounce homogenizer, and DNA was extracted from homogenized tissue with an Omniprep DNA extraction kit for high-quality genomic DNA (G-Biosciences), following the standard Omniprep base kit protocol with adjustments outlined in Mongue, Markee, et al. 2024 and Liesenfelt et al. 2025. This modified protocol, which has successfully generated chromosome-level assemblies of other insect species, included lysing tissue for 15 to 16 h overnight in an incubator at 56 °C. To increase DNA yield, we added 2 μL of 20 mg/mL Mussel Glycogen (Thermo Scientific) to each sample during the precipitation step. After Mussel Glycogen was added to each sample, we allowed DNA to precipitate for 1 h in a −20 °C freezer. In tandem, these modifications to the standard Omniprep protocol allowed us to maximize our DNA yields.

#### Quality control and sequencing

We used a Qubit 4 Fluorometer with Qubit dsDNA 1X Broad Range Sensitivity Assay Kit and a NanoDrop spectrophotometer to check the quantity and quality. We sent both DNA and frozen tissue from an additional 2 individuals to the Arizona Genomics Institute (Tucson, AZ, USA) for Pacific Biosciences (PacBio) HiFi sequencing on a Revio and for Illumina Hi-C library preparation and sequencing, respectively. Following sequencing of the male specimen, we subsequently repeated extraction and sequencing for a single female sample for sex chromosome identification. For this analysis, we sequenced extracted DNA as 150-bp paired-end Illumina reads with SeqMatic Incorporated (Fremont, CA, USA).

### Genome size estimation

To estimate the expected genome size, we used Jellyfish v.2.2.4 (Marçais and Kingsford 2011) to count 21-mers with the parameter -m 21. The resultant data were analyzed using custom scripts in RStudio v.4.2.0 (R Core Team 2020) to estimate both genome size and the coverage depth of homozygous regions.

### Genome assembly

#### Contig assembly from raw reads

We used the Hifiasm v.0.18 program (Cheng et al. 2021) to assemble our primary genome with the -l 3 parameter for more aggressive removal of haplotigs and the -hom-cov set from the results of the k-mer coverage discussed in the previous section. After primary assembly, we further purged remaining haplotigs from our assembly using the purge_dups pipeline (https://github.com/dfguan/purge_dups). We assessed the assembled genome for completeness with the hemipteran ortholog dataset (hemiptera_odb10) in BUSCO v.5.7.0 and for quality with Merqury v.1.1 (Rhie et al. 2020; Manni et al. 2021). We also ran Blobtools v.4.3.5 to check for any cobiont contaminants (Challis et al. 2020).

#### Hi-C scaffolding to resolve chromosomes

To bring our assembly to the chromosome level, we scaffolded our PacBio primary assembly with our Hi-C sequencing data. We used the Arima Hi-C pipeline (https://github.com/ArimaGenomics/mapping_pipeline) to align Hi-C reads to our primary assembly, as we have successfully done in other insects (Mongue et al. 2026). We first used YaHS v.1.1 (Zhou et al. 2022) to do automated scaffolding. Next, we generated a JBAT file of the initial scaffolded assembly, which we visualized as a contact map using Juicebox v.2.3.0 (Robinson et al. 2018) and manually curated misassemblies. Again, we assessed the curated genome for completeness with BUSCO. At this stage, we also checked for telomeric repeats using the insect AATCC motif (Stoianova et al. 2025) with tidk v.0.2.7 (Brown et al. 2025)

Finally, we realigned the raw male reads to the curated assembly to identify the X chromosome via coverage differences compared to the autosomes, following similar methods for other arthropods (Mongue and Baird 2024). To differentiate the X from the Y (if assembled), we separately aligned the female sequencing data to the genome. We then used SAMtools v.1.9 (Li et al. 2009) to sort alignments and calculate per-scaffold coverage. In males, the X and Y should have roughly half the coverage of the autosomes; in females, the X and autosomes should have equal coverage and any Y-linked sequences should have no coverage.

### Repeat masking

Repeat characterization and masking was done as a 2-step process. First, we used RepeatModeler v.2.0 (Flynn et al. 2020) to find de novo repeats; the “-LTRStruct” parameter was used to additionally find long terminal repeats. The results of this were run through RepeatMasker v.4.0.9 (Smit et al. 2019), which generated a soft-masked assembly and returned a summary of the identified repeats. This analysis was completed for *P. mystica,* as well as the publicly available chromosome-level genomes of *C. lectularius, Panstrongylus geniculatus, Rhynocoris fuscipes*, and *Triatoma dimidiata*, accessed from NCBI. The other taxa were taken through this step as part of gene annotation to perform synteny analyses described below.

### Gene annotation

Owing to limited tissue availability, we did not generate RNA-seq datasets for this study, opting instead to use the BRAKER3 annotation program (Gabriel et al. 2024) to annotate protein-coding genes from the soft-masked genome. As evidence, we used concatenated protein sequences from hemipteran BUSCOs. We conducted our analysis of the BRAKER-annotated genes with the hemiptera_odb10 database to assess completeness. We estimated completeness using only the longest transcript for each gene, which we parsed using a custom bash script.

### Phylogenetic methods

To determine the phylogenetic relationships between *P. mystica* and other members of Reduviidae, we downloaded existing annotated genomes for other assassin bugs and our outgroup, *C. lectularius*, as shown in Supplementary Table 4. To analyze the transcriptomic data, we assembled the transcriptomes using Trinity v.2.9.0 (Grabherr et al. 2011). To extract single-copy orthologs for use in phylogenetic inference, we directly ran BUSCO (Manni et al. 2021) on the genomes.

After this, we used a BUSCO phylogenomics pipeline to gather single-copy BUSCOs found in a minimum of 75% of our sample species and used FastTree v.2.1.11 (Price et al. 2010) to generate individual gene trees for each protein sequence in accordance with the Jones-Taylor-Thornton model of evolution (Jones et al. 1992) with the CAT model approximation for different rates at each site (Lartillot and Philippe 2004).

Next, we generated a consensus species tree using Accurate Species Tree EstimatoR's ASTRAL tool v.1.22 (Zhang et al. 2025) with the parameter “-R” for more subsampling and placement options, and used *C. lectularius* to root as the outgroup, as it is a known member of a separate family within the same infraorder (Cimicomorpha). We assessed confidence in the resulting tree with SH-aLRT/UFbootstrap values.

### Synteny analysis

To further explore the evolution of ambush and assassin bugs, we characterized shared synteny among species with chromosome-level assemblies using the MCScanX toolkit 1.0.0 (https://github.com/wyp1125/MCScanX). In brief, it utilizes NCBI's Basic Local Alignment Search Tool to conduct pairwise protein sequence comparisons to identify putative 1-to-1 orthologs between species, then uses genomic coordinates of those genes to map orthologous regions between genomes. To visualize the synteny between *P. mystica* and available chromosome-level assemblies, we used the SynVisio: Synteny Browser (https://github.com/kiranbandi/synvisio).

### Ortholog analysis

To determine the presence of salivary-protein orthologs, we used Proteinortho v.6.0 (Lechner et al. 2011), a tool that detects orthologous genes via sequence similarity. We compared the *P. mystica* gene models to the previously identified sialomes expressed by 2 predatory assassin bug proteomes (*Ectomocoris sp.* and *Oncocephalus sp.*) (Zdenek et al. 2024), 1 hematophagous assassin bug proteome (*Triatoma pallidipennis*) (Hernández-Vargas et al. 2017), 1 hematophagous assassin bug salivary gland transcriptome (*Rhodnius prolixus*) (Aguilera-Uribe et al. 2020) and 1 hematophagous heteropteran (*C. lectularius*) salivary gene set. The set of salivary genes used for comparison with *C. lectularius* was compiled through an NCBI gene search of *C. lectularius* with the keywords “salivary/saliva”, “venom”, and “sialome” (Supplementary Table 2). These additional species samples had their repeats modeled and masked in the same manner as our *P. mystica* sample.

The identification of salivary proteins in *Ectomocoris sp*., *Oncocephalus sp.*, and *T. pallidipennis* was taken from venom profiles described in Zdenek et al. 2024 and Hernández-Vargas et al. 2017. The salivary gland transcriptome of *R. prolixus* was not annotated in the original publication, so InterProScan 6, which generates predicted protein families for a list of provided sequences, was used to predict salivary protein functions with default parameters. The venom proteins described in these papers were searched against the annotated genome of *P. mystica*. Annotated protein families were then sorted into 17 categories: DNA replication/repair, housekeeping, hydrolase, kinases/phosphatases, non-serine proteases, oxidoreductases, protease inhibitor, protein degradation, signaling proteins, structural proteins, transposable elements, transcription/translation, transferases, transport proteins, ubiquitin system, venom proteins, and other. For proteins in the “venom proteins” category, or those having putative venom activity, toxin classification definitions (cytotoxic, hemotoxic, neurotoxic, digestive, and synergist) were taken from a review of the relevant literature and UniProt functional annotation (Valenzuela et al. 1998; Assumpção et al. 2008; Yang et al. 2008; Trevisan-Silva et al. 2010; Choo et al. 2012; Zdenek et al. 2024).

## Results and discussion

### Initial genome size estimates

We received PacBio HiFi long reads in 3 runs from the same sample and library; the first run yielded 20.52 Gb of read data, the second 17.66 Gb, and the third 13.05, for a total of 51.25 Gb (raw read data accessions found in Table 1). We found 2 peaks in our k-mer coverage plot at a sequencing depth of 14×, representing the heterozygous/hemizygous and homozygous coverage, respectively, and estimated the genome to be 741 Mb, with an average Merqury quality value (QV) of 59.2, well above the QV30 benchmark of 99.9% base accuracy.

**Table 1.** Assembly statistics of the primary HiFi assembly, the HiFi assembly with duplications purged, the Hi-C scaffolded assembly, and the final curated assembly.

Assembly Statistics	Primary HiFi assembly (Hifiasm)	HiFi assembly w/duplications purged (Hifiasm)	Hi-C scaffolded assembly (YaHS)	Final curated assembly
**Total length (bp)**	1,277,633,756	710,813,075	710,885,175	710,885,175
**Sequence count**	2,091	883	293	299
**N50**	1,615,564	1,958,841	47,149,494	47,149,494
**L50**	226	103	5	5
**GC content (%)**	31.56	31.54	31.54	31.54
**BUSCO completeness score (hemipteraodb10, *n* = 2,510)**	C:97.6% [S:12.0%, D:85.6%], F:0.6%, M:1.8%, n:2,510, E:1.8% 2,451 Complete BUSCOs (C) (of which 44 contain internal stop codons) 302 Complete and single-copy BUSCOs (S) 2,149 Complete and duplicated BUSCOs (D) 14 Fragmented BUSCOs (F) 45 Missing BUSCOs (M) 2,510 Total BUSCO groups searched	C:97.4% [S:93.6%, D:3.7%], F:0.8%, M:1.9%, n:2,510, E:2.5% 2,444 Complete BUSCOs (C) (of which 61 contain internal stop codons) 2,350 Complete and single-copy BUSCOs (S) 94 Complete and duplicated BUSCOs (D) 19 Fragmented BUSCOs (F) 47 Missing BUSCOs (M) 2,510 Total BUSCO groups searched	C:97.6% [S:93.9%, D:3.7%], F:0.6%, M:1.8%, n:2,510, E:2.7% 2,451 Complete BUSCOs (C) (of which 67 contain internal stop codons) 2,358 Complete and single-copy BUSCOs (S) 93 Complete and duplicated BUSCOs (D) 14 Fragmented BUSCOs (F) 45 Missing BUSCOs (M) 2,510 Total BUSCO groups searched	C:97.6% [S:93.9%, D:3.7%], F:0.6%, M:1.8%, n:2,510, E:2.7% 2,451 Complete BUSCOs (C) (of which 67 contain internal stop codons) 2,358 Complete and single-copy BUSCOs (S) 93 Complete and duplicated BUSCOs (D) 14 Fragmented BUSCOs (F) 45 Missing BUSCOs (M) 2,510 Total BUSCO groups searched

### Sequencing and assembly

Hi-C scaffolding confidently assembled *n* = 14 chromosomes, with scaffold_2 identified as the single X via coverage difference from the autosomes in males. The expected Y chromosome may be poorly assembled; we identified 9 scaffolds with no coverage in the female sample and non-zero coverage in the male. Of these, only 1 (scaffold_200) has the expected ∼½ autosomal coverage in males; the rest have very low coverage (Supplementary Table 1) and are likely junk sequences (eg error-containing sequences or repeats that differ between samples) or co-biont contaminants present in the assembled sample but not the resequenced female (eg scaffold_199). The best-supported Y-linked sequence is a mere 31 kbp with 17 annotated protein-coding genes. Expanding to include all apparently male-specific sequences, even those of dubious origin, yields 199,051 bp and a total of 46 genes. By either estimate, the Y is clearly highly degenerated compared to the X, which is the second largest chromosome at 84 Mb and contains 3,478 genes.

More generally, the inferred overall chromosome number is typical of other chromosome-level assemblies of assassin bug genomes (*n* = 12 to 15), with a key difference being that other sequenced assassin bugs have 2 distinct X chromosome pairs: X_1_ + X_2_ (Canizales-Silva et al. 2025; Kuznetsova et al. 2025). We placed 99.7% of the assembly within our 14 chromosomal scaffolds. In our final curation steps, we made 23 manual adjustments to our assembly using the Juicebox v.2.3.0 tool, including purging 6 duplications and correcting 17 misassemblies. Only adjustments deemed necessary to realign contigs into their correct chromosomal arrangement were made; this was determined through interaction frequency between regions in our assembly. Our final curated assembly received a BUSCO score of 97.6% completeness (93.9% single copy, 3.7% duplicated, 0.6% fragmented, and 1.8% missing) (Table 1).

Our estimated genome size of 741 Mb was greater than our measured genome size of 710 Mb. Despite having fewer chromosomal scaffolds, the size of *P. mystica*'s genome (710 Mb) is larger than *R. fuscipes* (*n* = 15, 620 Mb) (GCA_040020575.1). Though perhaps not surprising, as sometimes a lower chromosome count is associated with larger genome size, eg the cottony cushion scale, *Icerya purchasi,* has a genome size > 1 Gb contained in 2 massive chromosomes (Mongue, Ross, et al. 2024). Telomeric repeat search found short telomeres on all chromosomal scaffolds (minimum of 2 tandem repeats of AATCC), 4 scaffolds with at least 3 repeats, and only 1 scaffold, scaffold_7, with 4 telomeric repeats. Finally, we assessed evidence of co-biont contamination and found no large sources of contamination. A full description and Blob plot can be found in the supplement, but in short, we found very little potential contamination and removed a small set of low-coverage scaffolds that hit to Microsporidia and *Spiroplasma*, totaling roughly 300 kb of sequence.

### Phylogeny of Reduviidae

Using available genomic and transcriptomic resources, we explored the phylogenetic relationships between *P. mystica* and other reduviid species, representing 6 subfamilies (Fig. 1). The tree was rooted using *C. lectularius*, a non-reduviid hemipteran. We inferred the overall species trees through the use of the hemipteran ortholog dataset (hemiptera_odb10). We recovered *P. mystica* as sister to the other assassin bugs that were sampled in our BUSCO-based phylogenetic analysis. *P. geniculatus* and *Hospesneotomae protracta* rendered *Triatoma* paraphyletic, which has been recovered previously (Justi et al. 2014). This molecular evidence suggests that the reclassification of these species may be required, though it is outside the scope of this study.

**Fig. 1.**
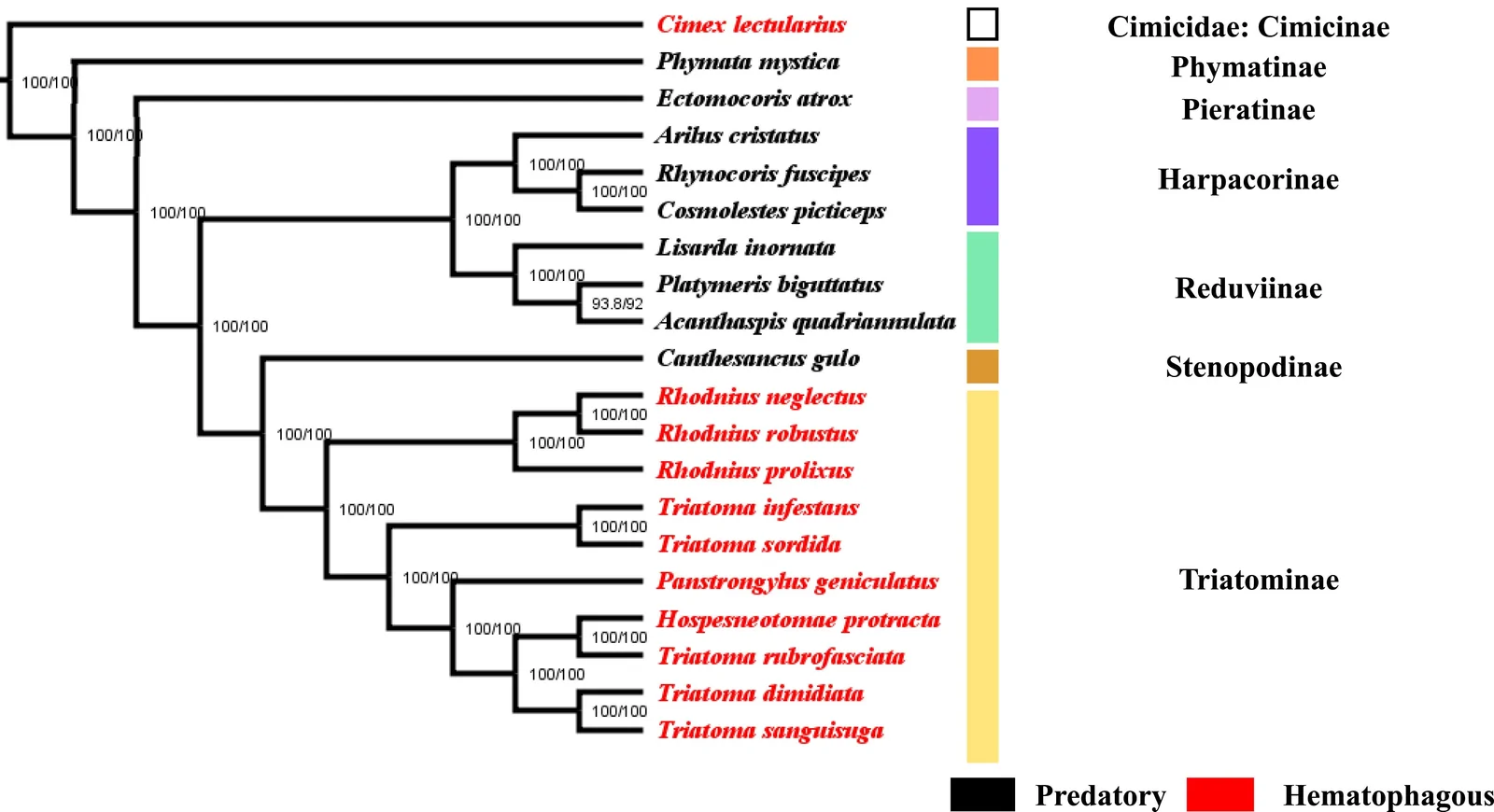
Cladogram of maximum likelihoods for species reported in this study. Support values are formatted as Shimodaira-Hasegawa-like approximate likelihood ratio test (SH-aLRT)/Ultrafast Bootstrap values and are shown at each node. Subfamily designations are listed to the right of their associated terminal(s). Hematophagous species terminals have their names in red.

### Repeat masking

Within our annotated assembly, repeat elements accounted for 58.85% of the sequence (418,374,926 bases). The full extent of repeat content can be seen in Table 2. *P. mystica* had the highest repeat content of any reduviid assembled to the chromosome-level, with other species having a repeat element content between 46.26 and 52.55%.

**Table 2.** Summary of the identified and masked repeats in the *Phymata mystica* genome.

Category	Count	Length (bp)	Percent
**Retroelements**	516,111	117,707,204	16.56%
**SINEs**	5,962	704,433	0.10%
**Penelope**	0	0	0.00%
**LINEs**	278,585	73,216,923	10.30%
**CRE/SLACS**	0	0	0.00%
**L2/CR1/Rex**	92,712	25,964,701	3.65%
**R1/LOA/Jockey**	30,801	7,748,315	1.09%
**R2/R4/NeSL**	19,188	5,453,623	0.77%
**RTE/Bov-B**	33,061	9,293,259	1.31%
**L1/CIN4**	5,233	840,251	0.12%
**LTR elements**	231,564	43,785,848	6.16%
**BEL/Pao**	3,115	2,717,414	0.38%
**Ty1/Copia**	1,303	1,038,681	0.15%
**Gypsy/DIRS1**	136,580	25,783,133	3.63%
**Retroviral**	90,400	14,220,918	2.00%
**DNA transposons**	335,595	74,823,119	10.53%
**hobo-Activator**	89,389	19,901,044	2.80%
**Tc1-IS630-Pogo**	108,908	23,570,544	3.32%
**En-Spm**	0	0	0.00%
**MULE-MuDR**	3,179	816,183	0.11%
**PiggyBac**	3,467	1,086,840	0.15%
**Tourist/Harbinger**	1,943	585,327	0.08%
**Other (Mirage, P-element, Transib)**	694	296,324	0.04%
**Rolling-circles**	282,023	67,169,590	9.45%
**Unclassified**	1,208,681	225,844,603	31.77%
**Total interspersed repeats**	—	418,374,926	58.85%
**Small RNA**	7,788	1,725,264	0.24%
**Satellites**	22,492	2,632,532	0.37%
**Simple repeats**	263,870	14,083,728	1.98%
**Low complexity**	32,170	1,719,575	0.24%

### Gene annotation

In total, we identified 26,760 protein-coding genes within our genome for *P. mystica* using the BRAKER3 tool. This annotation showed lower completeness than the genome assembly itself. Once parsed for the longest transcripts of each gene, BUSCO analysis scored the annotation with 92.5% completeness (S:88.8%, D:3.7%, 2.5% fragmented, and 1.0% missing). *P. mystica* had a larger genome compared to the other predatory reduviid with a chromosome-level sequence, *R. fuscipes*, with only 16,189 protein-coding genes. *P. mystica*'s genome shared a similar number of protein-coding genes to the 2 hematophagous reduviids with chromosome-level assemblies, 21,125 genes in *T. pallidipennis* and 24,047 in *P. geniculatus*. Future generation of an RNA-seq dataset will help refine and improve gene predictions for *P. mystica*.

### Synteny

Our synteny plot found multiple fission events, including 1 involving the X chromosome after the split between *P. mystica* and higher Reduviidae (Fig. 2). Despite this change, both parts of the formerly single X are sex-linked in *P. geniculatus*, which suggests that the X_1_X_2_ system is conserved in *Rhynocoris* and *Triatoma*. Several other crossovers, fission, and fusion events were found to have occurred in the autosomes in the splits between Phymatinae (*P. mystica*) and Harpactorinae (*R. fuscipes*), and Harpactorinae and Triatominae (*P. geniculatus).* We found no crossovers, fission, or fusion events in the split between *P. geniculatus* and *T. pallidipennis*, both members of Triatominae.

**Fig. 2.**
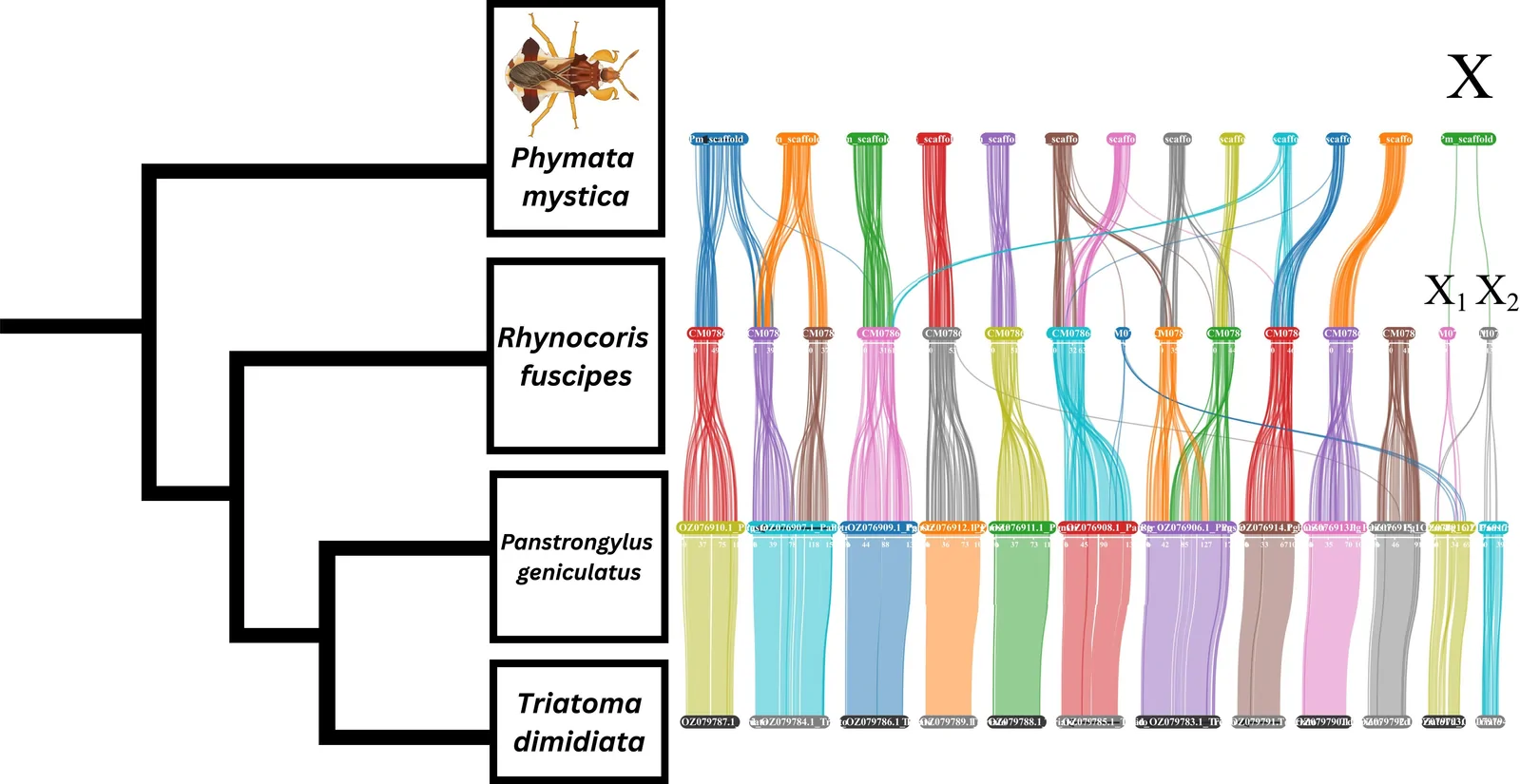
Synteny between chromosome-level assemblies of Reduviidae, showing the fission of the X chromosome and other autosomes after the split between *Phymata mystica* and the higher Reduviidae. The chromosomes of each of the 4 species shown are illustrated as colored tabs; lines emanating from these tabs, top-down, show the movement of genes between chromosomes. The X chromosome is the rightmost tab in *P. mystica,*, whereas the 2 rightmost tabs represent the X chromosome, which is split into X_1_ and X_2_. The X chromosome is only putatively identified in *P. mystica* and *P. geniculatus. P. mystica is* illustrated by TL.

### Ortholog conservation

Our ortholog analysis found 885 unique proteins conserved across the salivary proteomes of *Ectomocoris sp., Oncocephalus sp*., and *T. pallidipennis*, the annotated transcriptome of *R. prolixus*, and the salivary gene set from *C. lectularius* with the full annotation of *P. mystica*. While orthogroups inferred missing from *P. mystica* may be partly attributable to the imperfect annotation, those that were recovered as conserved are informative. Of the 885 unique proteins found in our comparative search of the *P. mystica* genome, 109 were unidentifiable to the family level, 54 were classified as proteins with venom activity, 53 as transferases, 42 as proteins with transcription/translation activity, 35 as hydrolases, 35 as transport proteins, 29 as kinases/phosphatases, 28 as oxidoreductases, 27 as housekeeping proteins, 22 as TEs, 20 as signaling proteins, 19 as non-serine proteases, 10 as structural proteins, 10 as ubiquitin system, 8 as protein degradation, 7 as proteins involved in DNA replication/repair, 3 as protease inhibitors, and 372 as having other annotated functions not in other listed categories. Comparative profiles of the searched salivary proteomes of *Ectomocoris sp.*, *Oncocephalus sp.*, and *T. pallidipennis*, the annotated transcriptome of *R. prolixus*, and the salivary gene set of *C. lectularius* can be seen in Fig. 3a.

**Fig. 3.**
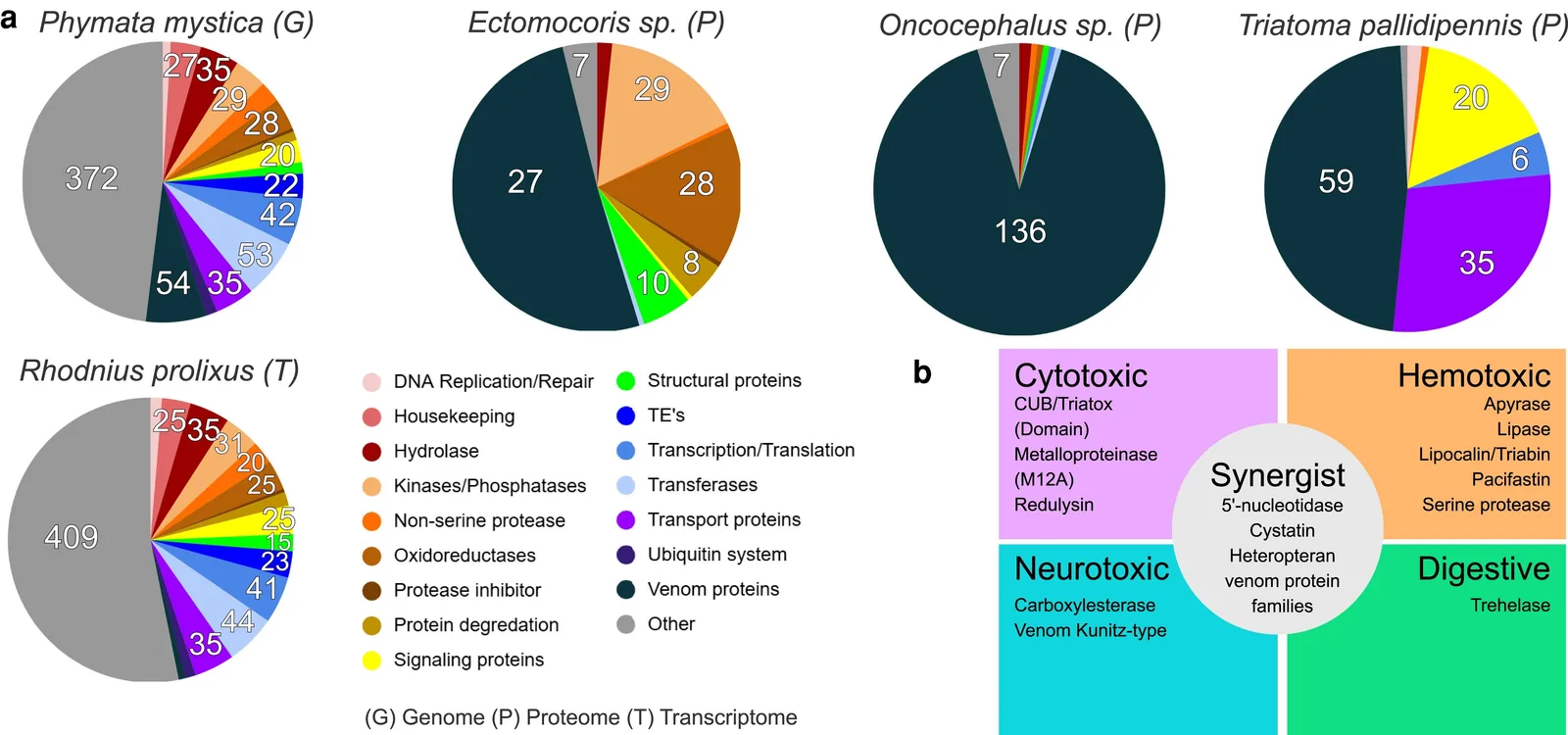
a) Salivary protein profile of *Phymata mystica* inferred from direct orthology, compared with the full venom and salivary profiles of related species. The profile inferred for *P. mystica* represents the conserved/ancestral state. b) Protein families inferred to be produced by *P. mystica* from genetic data, grouped by documented venomic effect from arthropod samples. Venom class definitions come from a review of relevant literature and UniProt functional annotation as described in the methods section. Lipocalin is present in higher amounts in the inferred *P. mystica* profile than in other predatory species. All venom effect classifications are found in *P. mystica*.

We found representatives from 14 venom protein families conserved in *P. mystica*. A breakdown of protein families by predicted venomic effect can be seen in Fig. 3b. Of the putative venom proteins found to have orthologs in the *P. mystica* genome, the largest group represented in our analysis is proteases, mainly serine proteases with some metallopeptidases. Of the serine proteases detected, 8 contained a CUB domain. There is a considerable amount of orthology with proteins in ungrouped heteropteran venom families, including families 1, 2, 3, and 6. The presence of serine proteases and heteropteran venom family proteins in the venom of *P. mystica* is consistent with the sialome of other predatory reduviids (Zdenek et al. 2024). We found 2 orthologs of lipocalin (Zdenek et al. 2024), the primary component of *T. pallidipennis* venom, in *P. mystica* from both *C. lectularius* and *T. pallidipennis*. A comprehensive list of conserved venoms can be found in Supplementary Table 3. In summary, in spite of rough initial annotation, we found all classes of assassin bug venom groups in the ambush bug, demonstrating that these proteins evolved very early in the family, most likely present in the most recent common ancestor to the reduviids. In contrast, we found more lipocalin family proteins in *P. mystica* than in *Ectomocoris sp.* and *Oncocephalus sp.*, showing changes to venom composition in the outgroup to all other Reduviidae (Phymatinae) despite overall similar content.

## Conclusions

We report a chromosome-level genomic assembly of *P. mystica*, a venomous ambush bug in the understudied Phymatinae. This assembly provides the essential resources for further genetic research into this subfamily. Our curated assembly, annotation, and repeat data provide a means of comparison to other members of the family Reduviidae. The record of conservation of venoms produced by this study serves as a jumping-off point for more complete venom profiles to be generated from *P. mystica* and further comparisons to elucidate the evolution of venoms across assassin bugs.

## Supplementary Material

jkag244_Supplementary_Data

## Data Availability

All relevant data will be publicly accessible through the National Library of Medicine: National Center for Biotechnology Information (NCBI). Raw sequence data and accession numbers are available in Supplementary Table 4. Annotation, along with analysis scripts, can be found on the GitRepo: https://github.com/ethangrebler/phymata_genome, and full venom conservation details are provided in the supplementary file (Supplementary Table 3). Supplemental material is available at G3 online. Sequencing data can be found on NCBI with the following accessions: SRR36928797 for PacBio HiFi, SRR36928795 for Hi-C, and SRR36928796 for the female short reads. The final curated assembly can be found on NCBI under the accession: JBVFRP000000000. Supplemental material available at G3 online.
