# Supplementary material for: Genome report: genome sequence of Phymata mystica (Evans), an ambush bug
Source: G3 (Bethesda). 2026 Aug 28;16(11):jkag244. doi: 10.1093/g3journal/jkag244 (PMC13732435; doi:10.1093/g3journal/jkag244)
Supplement: Supplementary file 1 [file jkag244_supplementary_data.pdf]

Supplemental Materials

Co-biont screening with Blobtools

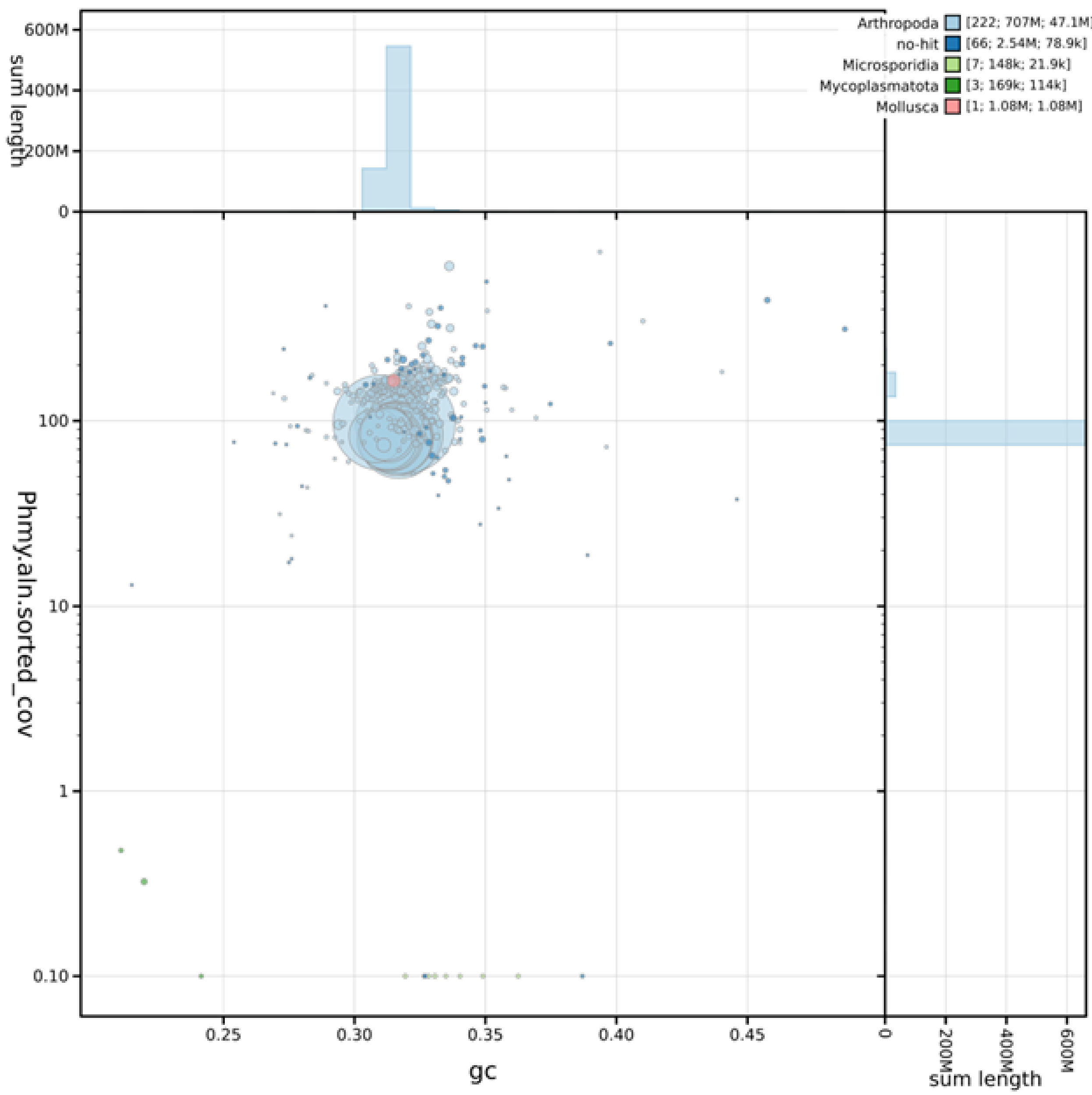

Figure S1. Blobtools plot showing coverage (y axis), GC content (x axis), and BLAST hits for scaffolds (colored circles) of the *P. mystica* assembly. Taxonomic assignments are made via BLAST hit to the phylum level and circle sizes are proportional to the length of the scaffold.

We screened the assembled genome for putative contaminants based on the Blobtools pipeline. We found a few small scaffolds that hit to fungi (Microsporidia) and bacteria (Microplasmatota), which also had lower coverage and in some cases distinct GC content from the core genome (Figure S1, bottom and bottom left). These represented the most likely contaminants and were removed from the assembly. A single scaffold hit to Mollusca as well, but given the singular hit, and lack of difference in coverage or GC content from the rest of the genome, we considered it as more likely to be a false positive than true contaminant and left in the assembly.

Table S1. Abridged coverage based inference of sex-linked sequences. For brevity, we show only two autosomal scaffolds to demonstrate the pattern, but all omitted scaffolds had autosomal coverage patterns.

Scaffold	Length (bp)	Male coverage depth	Female coverage depth	Chromosome Inference
scaffold_1	92,333,288	25	33	Autosome
scaffold_2	84,087,695	15	34	X chromosome
scaffold_3	60,226,300	26	33	Autosome
...		...	...	...
scaffold_199	31,794	3	0	Putative co-biont
scaffold_200	31,179	17	0	Putative Y chromosome
scaffold_209	24,421	8	0	Putative junk or Y
scaffold_216	21,944	3	0	Putative junk
scaffold_217	21,297	4	0	Putative junk
scaffold_226	18,604	4	0	Putative co-biont
scaffold_234	17,291	1	0	Putative co-biont
scaffold_237	16,608	2	0	Putative junk
scaffold_239	16,093	6	0	Putative junk

Table S2. Salivary proteins of *Cimex lectularius* obtained from uniprot based on functional annotation, that were searched against the annotated *Phymata mystica* genome.

Accession	Gene / Protein Name
D1FPL8	14-3-3 protein zeta

D1FPL9	14-3-3 protein zeta
D1FPM4	60S ribosomal protein L11
D1FPN7	ADP-ribosylation factor
D1FPN0	ADP-ribosylation factor-like 5a
O96559	Apyrase
A0A8I6RDY3	BHLH domain-containing protein
D1FPL1	Bis(5'-nucleosyl)-tetraphosphatase
D1FPM3	Bis(5'-nucleosyl)-tetraphosphatase
D1FPM5	Bis(5'-nucleosyl)-tetraphosphatase
D1FQG8	Carboxylic ester hydrolase
A0A1V0BZA3	Cytochrome c oxidase subunit 1
A0A8I6RF1	DE-cadherin
A0A8I6TEX8	DE-cadherin
A0A8I6RES3	dTCF
A0A8I6RGC2	dTCF
A0A8I6RJ27	dTCF
A0A8I6TCV5	dTCF
D1FPM1	E3 ubiquitin ligase
D1FPL2	F-ATPase delta subunit
A0A8I6RGK1	HMG box domain-containing protein
D1FPL5	Large ribosomal subunit protein mL62
D1FPL7	Large ribosomal subunit protein P1
D1FPL3	Large ribosomal subunit protein uL1
D1FPP2	M phase phosphoprotein 6
O76745	Nitrophorin Cim l
A0A8I6RAV1	Peptidase M12B domain-containing protein
A0A8I6RDG4	Peptidase M12B domain-containing protein
A0A8I6REJ0	Peptidase M12B domain-containing protein
D1FPM6	peptidylprolyl isomerase
D1FPN3	Phosphate carrier protein, mitochondrial
D1FPN1	proteasome endopeptidase complex
A0A8I6TFJ5	Ras-related protein Rac1
D1FPN9	Ras-related small GTPase rho type

A0A8I6RDJ2	receptor protein-tyrosine kinase
A0A8I6RES9	receptor protein-tyrosine kinase
A0A8I6SG88	receptor protein-tyrosine kinase
A0A8I6RDM0	Salivary lipocalin
A0A8I6RWT8	Salivary lipocalin
D1FPM0	Salivary nitrophorin
A0A8I6S2J2	Salivary OBP
A0A8I6TDJ0	Salivary OBP
D1FPL4	Salivary OBP
D1FPM2	Salivary OBP
D1FPM7	Salivary OBP
A0A8I6RA51	Salivary secreted peptide
A0A8I6RXG9	Salivary secreted peptide
A0A8I6TCT9	Salivary secreted peptide
A0A8I6TGI3	Salivary secreted peptide
A0A8I6TKA4	Salivary secreted peptide
A0A8I6R8W7	Salivary secreted protein
A0A8I6S2C6	Salivary secreted protein
A0A8I6S2L3	Salivary secreted protein
A0A8I6S935	Salivary secreted protein
A0A8I6SIE0	Salivary secreted protein
D1FPM8	Signal peptidase complex subunit 2
D1FPP1	Small integral membrane protein 14
D1FPF3	Small ribosomal subunit protein eS6
D1FPN4	Small ribosomal subunit protein uS9
D1FPM9	Translation machinery-associated protein 7 homolog
D1FPP3	Tubulin-specific chaperone A
A0A8I6SQK7	Tumor necrosis factor alpha-induced protein 8-like
A0A8I6SSI8	Tumor necrosis factor alpha-induced protein 8-like
A0A8I6S1E0	Tyrosine-protein kinase
A0A8I6SIF0	Tyrosine-protein kinase
A0A8I6SK60	Tyrosine-protein kinase
A0A8I6SMZ8	Tyrosine-protein kinase

A0A8I6TKM1	Tyrosine-protein kinase
A0A8I6TMH2	Tyrosine-protein kinase
D1FPL6	Ubiquitin-fold modifier 1

Table S3. Venom orthologs found in the annotated *Phymata mystica* genome, along with the amount of orthologous proteins found, their sourced organism and sourced publication.

Protein Family	Number of Orthologs	Sourced Species	Sourced Publication/Uniprot Accession(s):
Serine protease	10	*Ectomocoris sp., Oncocephalus sp.*	Zdenek *et al.* 2024
Metallopeptidase	3	*Ectomocoris sp., Oncocephalus sp., T.pallidipennis*	Hernández-Vargas *et al.* 2017<br>Zdenek *et al.* 2024
Carboxylesterase	2	*Ectomocoris sp.*	Zdenek *et al.* 2024
Heteropteran venom family 3	2	*Ectomocoris sp.*	Zdenek *et al.* 2024
Lipase	2	*Ectomocoris sp., Oncocephalus sp.*	Zdenek *et al.* 2024
Liopcalin	2	*C.lectularius, T.pallidipennis*	A0A8I6RDM0<br>Hernández-Vargas *et al.* 2017
5' nucleosidease	1	*T.pallidipennis*	Hernández-Vargas *et al.* 2017
Apyrase	1	*C.lectularius*	O96559
Heteropteran venom family 1	1	*Ectomocoris sp., Oncocephalus sp.*	Zdenek *et al.* 2024
Heteropteran venom family 2	1	*Oncocephalus sp.*	Zdenek *et al.* 2024
Heteropteran venom family 6	1	*Ectomocoris sp.*	Zdenek *et al.* 2024
Pacifastin	1	*Oncocephalus sp.*	Zdenek *et al.* 2024
Redulysin	1	*Ectomocoris sp., Oncocephalus sp.*	Zdenek *et al.* 2024
Trehelase	1	*Ectomocoris sp.*	Zdenek *et al.* 2024
Venom Kunitz-type	2	*Rhodnius prolixus*	Aguilera-Uribe *et al.* 2020

Table S4. BUSCO Stats for genomic and transcriptomic data used in this study, along with their accession numbers and documented karyotype. If a venom profile was accessed for a species, the publication in which the profile was noted is documented.

Species Name	Subfamily	Data Type	BUSCO Stats	Accession	Karyotype (n)
Cimex lectularius	Cimicidae: Cimicinae	genome	C:94.3%[S:89.9%,D:4.5%],F:0.8%,M:4.9%,n:2510,E:1.9%	GCA_049903775.1	15
Panstrongylus geniculatus	Triatominae	genome	C:99.0%[S:97.9%,D:1.0%],F:0.6%,M:0.4%,n:2510,E:3.9%	GCA_964188295.1	10 + X1X2
Phymata mystica	Phymatinae	genome	C:97.6%[S:93.9%,D:3.7%],F:0.6%,M:1.8%,n:2510,E:2.7%	(This Paper)	13+X
Rhodnius prolixus	Triatominae	genome	C:98.6%[S:98.0%,D:0.6%],F:0.3%,M:1.2%,n:2510,E:2.3%	GCA_049639745.1	
Rhynocoris fuscipes	Harpactorinae	genome	C:99.0%[S:95.8%,D:3.1%],F:0.3%,M:0.7%,n:2510,E:4.0%	GCA_040020575.1	15
Triatoma dimidiata	Triatominae	genome	C:98.8%[S:97.4%,D:1.4%],F:0.6%,M:0.7%,n:2510,E:4.0%	GCA_964198125.1	13
Triatoma infestans	Triatominae	genome	C:91.4%[S:89.8%,D:1.6%],F:2.7%,M:5.9%,n:2510,E:4.2%	GCA_011037195.1	
Triatoma sanguisuga	Triatominae	genome	C:99.1%[S:97.8%,D:1.3%],F:0.4%,M:0.4%,n:2510,E:3.8%	GCA_041753995.1	
Acanthaspis quadriannulata	Reduviinae	transcriptome	C:67.6%[S:43.7%,D:23.9%],F:7.4%,M:25.0%,n:2510	SRR13844089	
Arilus cristatus	Harpactorinae	transcriptome	C:86.0%[S:50.5%,D:35.5%],F:3.3%,M:10.8%,n:2510	SRR1821899	
Canthesancus gulo	Stenopodainae	transcriptome	C:70.2%[S:48.3%,D:21.9%],F:5.8%,M:24.1%,n:2510	SRR13844088	
Cosmolestes picticeps	Harpactorinae	transcriptome	C:88.8%[S:51.0%,D:37.9%],F:3.3%,M:7.9%,n:2510	SRR13844066	
Ectomocoris atrox	Peiratinae	transcriptome	C:92.3%[S:44.2%,D:48.1%],F:1.8%,M:5.9%,n:2510	SRR13844077	
Hospesneotomae protracta	Triatominae	transcriptome	C:91.8%[S:38.4%,D:53.5%],F:2.9%,M:5.3%,n:2510	SRR13844033	
Lisarda inornata	Salyavatinae	transcriptome	C:85.2%[S:47.9%,D:37.3%],F:3.6%,M:11.2%,n:2510	SRR13844055	
Platymeris biguttatus	Reduviinae	transcriptome	C:86.3%[S:45.1%,D:41.2%],F:2.7%,M:11.0%,n:2510	SRR17828248	
Rhodnius neglectus	Triatominae	transcriptome	C:90.8%[S:34.4%,D:56.5%],F:3.7%,M:5.4%,n:2510	SRR15602396	
Rhodnius robustus	Triatominae	transcriptome	C:89.1%[S:48.4%,D:40.6%],F:3.7%,M:7.2%,n:2510	ERR1315272	
Triatoma rubrofasciata	Triatominae	transcriptome	C:91.3%[S:43.3%,D:47.9%],F:2.6%,M:6.1%,n:2510	SRR30791502	
Triatoma sordida	Triatominae	transcriptome	C:68.9%[S:42.2%,D:26.7%],F:5.5%,M:25.6%,n:2510	SRR15218234	
